# CHANGES IN SOCIOECONOMIC STATUS ACROSS THE LIFE COURSE AND DEMENTIA ONSET IN JAPAN GERONTOLOGICAL EVALUATION STUDY

**DOI:** 10.1093/geroni/igz038.686

**Published:** 2019-11-08

**Authors:** Ryoto Sakaniwa, Ryoto Sakaniwa, Kokoro Shirai, hiroyasu iso, Katsunori Kondo

**Affiliations:** 1 Osaka University, Osaka University, Japan; 2 Graduate School of Medicine Osaka University, osaka, Osaka, Japan; 3 osaka university graduate school of medicine, osaka, Osaka, Japan; 4 Center for Preventive Medical Sciences, Chiba University, Chiba, Chiba, Japan

## Abstract

Socioeconomic status (SES) is associated with dementia onset, but its transition throughout the life-course is poorly understood. In a prospective cohort of 40,041 participants, aged over 65 years without dementia, we identified ten optimal classifications of life-course SES transitions and their associated impact on dementia. Our results showed a clear significant dose-response pattern with the highest risk of dementia from 1) impoverished SES throughout, 2) impoverished childhood SES with normal adult SES, 3) impoverished adult SES with self-employed in adulthood, 4) high educated with poor adult SES, 5) never having a job, 6) low educated but high SES (self- employed), 7) technician, 8) high educated with poor adult SES, 9) average SES throughout, and 10) high SES throughout. These results suggest that life-course SES history is strongly associated with dementia risk and did not interact with single scale measurement. Further causal pathways and theories are relevant for disease modification.

